# Simulation-based curriculum development: lessons learnt in Global Health education

**DOI:** 10.1186/s12909-020-02430-9

**Published:** 2021-01-07

**Authors:** Rasha D. Sawaya, Sandra Mrad, Eva Rajha, Rana Saleh, Julie Rice

**Affiliations:** 1 Department of Emergency Medicine, The American University of Beirut Medical Center, Beirut, Lebanon; 2 Department of Emergency Medicine, The University of Texas MD Anderson Cancer Center, Houston, TX USA; 3 Department of Emergency Medicine, Johns Hopkins University School of Medicine, 1830 E. Monument Street, Baltimore, MD 21205 USA

**Keywords:** Simulation curriculum, Education in low resource settings, Curriculum development

## Abstract

**Background:**

Simulation based medical education (SBME) allows learners to acquire clinical skills without exposing patients to unnecessary risk. This is especially applicable to Emergency Medicine training programs where residents are expected to demonstrate proficiency in the management of time critical, low frequency, and highly-morbidity conditions. This study aims to describe the process through which a SBME curriculum was created, in a limited simulation resource setting at a 4-year Emergency Medicine (EM) residency program at the American University of Beirut Medical Center.

**Methods:**

A case-based pilot simulation curriculum was developed following Kern’s 6 step approach to curriculum design. The curricular objectives were identified through an anonymous survey of the program’s residents and faculty. Curriculum outcomes were assessed, and the curriculum was revised to address curricular barriers. Evaluations of the revised curriculum were collected during the simulation sessions and through a whole revised curriculum evaluation at the end of the first year of its implementation.

**Results:**

14/20 residents (70%) and 8/8 faculty (100%) completed the needs assessment from which objectives for the pilot curriculum were developed and implemented through 6 2-h sessions over a 1-year period. Objectives were not met and identified barriers included cost, scheduling, resources, and limited faculty time. The revised curriculum addressed these barriers and 24 40-min sessions were successfully conducted during the following year. The sessions took place 3 at a time, in 2-h slots, using the same scenario to meet the objectives of the different learners’ levels. 91/91 evaluations were collected from participants with overall positive results. The main differences between the pilot and the revised curricula included: a better understanding of the simulation center resources and faculty’s capabilities.

**Conclusion:**

Simulation-based education is feasible even with limited-resources. However, understanding the resources available, and advocating for protected educator time are essential to implementing a successful EM simulation curriculum.

**Supplementary Information:**

The online version contains supplementary material available at 10.1186/s12909-020-02430-9.

## Background

Simulation based medical education (SBME) is a highly desired component of Emergency Medicine (EM) residency training programs as it allows learners to develop necessary knowledge, skills, and attitudes without exposing patients to unnecessary risk [1, 2]. This is especially important for specialties where learners are expected to demonstrate proficiency in the management of time-critical, low-frequency, and highly-morbidity conditions. With simulation, educators can provide a minimum number of simulated experiences during training to ensure exposure, while also preparing residents to fully participate in rare clinical experiences when they occur.

SBME has been shown to improve learner’s performance in both simulated and clinical settings [3–8]. Improvements in performance are commonly noted in the areas of technical skill development, trauma management, crisis resource management, and resuscitation skills training [3, 6, 9], all essential to the EM provider. Simulation-based training has also been shown to be superior to problem-based learning when teaching critical assessment and management skills [10]. These characteristics make the addition of a robust SBME component to an EM residency curriculum highly desirable for both faculty and residents.

As an educational method, one of the biggest challenges to implementing SBME is the cost. The combination of technology and the faculty time required for many experiential learning opportunities make simulation-based learning one of the more resource intensive educational methods available to educators [11]. Facilitation of simulation-based activities requires faculty training in debriefing and simulator logistics. This can pose a significant barrier when developing simulation based educational content to accompany a residency curriculum for the first time.

Our challenge was to create a SBME curriculum for an EM residency program located in the Middle East with limited simulation technology and faculty resources. This report describes the creation and implementation of a curriculum designed to complement existing educational programing at an academic EM program. We utilized Kern’s 6 step approach to curriculum design, which has been previously modified for the development of simulation programs [12]. Instead of a ‘stand-alone’ curriculum, SBME was incorporated into the existing resident educational programing as suggested by best practices for simulation program development [13].

During curriculum development, we confronted many barriers. These included educational activity planning for medical trainees whose schedules are variable, creating content to meet objectives for several different levels of trainees’ experience, and optimizing the use of limited faculty time. We hope our description of curriculum development methods, curriculum revisions, and the lessons learned during its implementation can serve as a guide for future EM educators who hope to incorporate SBME into their existing academic programs.

## Objective

Our aim was to create a SBME curriculum that complements the existing curriculum at an academic 4-year EM residency program with limited faculty and simulator resources in Beirut, Lebanon.

## Methods

This study was determined to be exempt from review by the Johns Hopkins and the American University of Beirut (AUB) Institutional Review Boards.

The simulation curriculum was developed based on the model presented by Kern et al. in Curriculum Development for Medical Education: A Six-Step Approach [14].

The six steps include:

1. Problem identification and general needs assessment
2. Targeted needs assessment
3. Goals and objectives
4. Educational strategies
5. Implementation
6. Evaluation and feedback

A pilot curriculum was developed according to the Kern’s methodology and in accordance with best practices for simulation program development [15]. After review of curricular outcomes, appropriate adjustments to curriculum design were made in order to optimize resource utilization and curriculum impact following a modified-Kern method. We will call this the revised curriculum.

### Needs assessment (KERN step 1 and 2)

General and targeted needs assessments were conducted through an anonymous survey of the EM program residents and faculty. A group of EM residents who attended weekly education conference were also interviewed as a convenience sample. Findings were supported by observations during clinical shifts from the research team. The results were discussed with education leadership and agreed to as appropriate targets for our SBME curricula.

An inventory of faculty educator availability and simulation resources was also performed which included access to one high technology manikin simulator, a simulation operations manager and a local faculty member interested in developing skills as a simulation-based educator.

### Goals and objectives (KERN step 3)

Goals and objectives were developed for each postgraduate level based on target areas identified by the needs assessment. Objectives were correlated with the Accreditation Council for Graduate Medical Education (ACGME) EM milestones by learner level.

### Educational strategies (KERN step 4)

Given the available resources and learning objectives, we chose to implement a case-based curriculum which incorporated repetitive practice and both deliberate and reflective feedback.

### Implementation (KERN step 5)

The curriculum was planned as a phase-in model where a local faculty member would schedule and incorporate modules throughout the year both in and outside planned education weekly conference time. Chief residents were tasked with scheduling resident participants outside of clinical time when necessary.

### Evaluation (KERN step 6)

Curriculum outcomes were assessed at the end of the 1st year of implementation and reviewed by study authors. These included number of sessions, number of participants and percent of curriculum achieved. Resources and time constraints of both participants and faculty were reviewed and discussed with local faculty and residents. The curriculum was then revised in response to identified curricular barriers.

## Results

### Needs assessment (KERN 1 and 2)

Fourteen residents (70%) completed the initial anonymous needs assessment survey (See Additional file 1). Several key areas for residency curriculum improvement were identified by EM residents and faculty members including building differential diagnosis, critical care resuscitation, communication and team leadership. Highly desired clinical topics and procedures were identified in resident and faculty surveys. 85% of those surveyed stated that they would be willing to attend simulation-based activities outside of the regularly scheduled weekly educational conference time.

The eight faculty surveyed (100%) identified “more practice with communication skills in clinical area” and “more practice taking care of acutely ill adult patients” as important skills that need improvement among junior and senior residents. They also reported that “more practice building differential diagnosis for patient presentations” was important for junior resident improvement and “more practice with resuscitation team leadership” was important for senior resident improvement. Areas of low reported confidence by junior and senior residents were corroborated by direct observation during clinical shifts.

### Goals and objectives (KERN 3)

Goals and objectives were created based on identified curriculum gaps (Additional file 2). Objectives for junior residents focused on history gathering, formulation of differential diagnosis and basic patient stabilization. Senior resident goals included advanced resuscitation, team leadership, communication and task switching.

### Educational strategies and program implementation (KERN 4/5)

#### Pilot curriculum

A summary of educational strategies for the pilot curriculum can be found in Table 1. Given the limited faculty resources, we planned to phase in a ‘train the trainer’ program where senior residents learned the basics of SBME facilitation in order to teach the junior resident curriculum. Senior residents would then receive faculty-led modules which would allow for more dynamic and challenging learning experiences for advanced learners.

**Table 1:** Barriers to implementation of the pilot curriculum and revisions implemented

	Barriers	Revisions
**Space**	• One simulation center for both the faculty of Medicine and Nursing at AUB opening on weekdays between 8 am and 4 pm	• We developed a close professional relationship with the Simulation Coordinator, with professionalism and following a regular schedule which allowed us regular access to the space
**Equipment**	• 1 adult and 1 pediatric manikin (could not accommodate mass casualty scenarios or multiple simultaneous activities)	• Adjusted scenarios to maximize use of the available manikins and other equipment
**Personnel**	• Only one simulation coordinator • Only 1 local faculty member facilitating the simulation activities	• Two Emergency physicians (in addition to the coordinator) with previously protected education time took over the simulation activities
**Time**	• Variable clinical schedules of EM trainees: make scheduling individual and small group modules off-time difficult • Limited faculty protected education time to implement curriculum	• Inclusion of simulation within the weekly resident conference • Use of published simulation scenarios • Using the same scenario and adapting its complexity to meet the different objectives according to trainees’ level of experience
**Administration**	• Limited stakeholder buy-in • Scheduling residents during off-hour required a significant amount of faculty and/or chief resident administrative time	• Involvement of EM educational leadership: EM residency associate program director • Inclusion of SBME in weekly conference minimized curriculum administration time for faculty and chief residents and ensured resident’s availability • Department leadership buy-in: funded one faculty sim training course

*EM* Emergency Medicine

*SBME* Simulation based medical education

### Evaluation/outcomes (KERN 6)

#### Pilot curriculum

Of the planned 30 2-h SBME sessions, only 6 took place: 5 during the weekly education conference (4 by a visiting US faculty and 1 by local faculty) and 1 outside of conference time. Given the difficulty with curriculum execution, faculty and facility resources were revisited and a list of barriers to implementation was identified (See Table 1).

#### Revised curriculum

Curriculum education and implementation strategies of the pilot were reviewed, and a revised curriculum was created to address local SBME resource constraints (see Table 2).

**Table 2:** Pilot and Revised Curriculum Description

Kern Steps	Pilot Curriculum	Revised Curriculum
**Kern 1 and 2: Needs Assessment**	• Faculty and resident survey which **focused on learner needs** with the following key components: building differential diagnoses, critical care resuscitation, communication and team leadership	• **Focused on resources** (based on barriers identified in the pilot curriculum and lessons learnt)
**Kern 3: Goals and Objectives**	• Junior residents: history gathering, formulation of differential diagnosis and basic patient stabilization • Senior residents: advanced resuscitation, team leadership, communication and task switching	• Unchanged
**Kern 4: Educational Strategies**	• 4-year curriculum • Individual (1, 2) and small (3, 4) group 2-h sessions • Faculty time: Thirty 2-h sessions in a year • Most sessions during resident off-duty hours • Train the trainer program (for PGY3) where seniors will eventually implement sessions • Session specific objectives	• 2- year curriculum • Medium (4, 5) group 40-min sessions • Residents grouped by PGY level • Faculty time: Monthly (12) 2-h blocks of time • During resident weekly conferences • 40-min modules, repeated 3 times (2-h blocks) • Modules’ objectives and complexities were tailored to the trainees’ level
**Kern 5: Implementation**	• 1 visiting faculty and 1 local faculty with limited protected time for simulation • Six 2-h sessions took place: - 5 for seniors during the weekly education conference (4 by a visiting and 1 by local faculty) - 1 for junior residents outside of weekly education conference	• 2 local faculty with protected time for resident education • Twenty-four 40-min sessions took place over 8 weekly conferences • Inclusion of nurses in the scenarios to increase credibility
**Kern 6: Evaluation/Outcomes**	• Poor curriculum feasibility, barriers recognized (Table 2) • No formal evaluations of modules due to limited implementation • Unable to implement the “train the trainer” program due to resident time constraints	• Session evaluations completed after each session and end of year curriculum evaluation Positive feedback received on the individual modules and on the curriculum as a whole

*min* minute

*PGY* Postgraduate year

During the second year of the curriculum, we planned for 27 40-min simulation sessions, and successfully implemented 24 (88.9%). Resident session evaluations were collected after each simulation (Additional file 3) as well as a curriculum evaluation after 1 year of the revised curriculum (Additional file 4). Changes based on end of session feedback were incorporated when feasible into the following sessions throughout the year. However, due to resource constraints we could not adjust the number of residents per session or the duration of the sessions, a recurring comment in the feedback of residents. Overall, the curriculum received positive feedback. Details of EM residents’ responses are found in Additional file 5.

## Discussion

Implementing SBME into residency education programs comes with many challenges. Among the primary barriers to SBME implementation in the EM residency program at the AUB were faculty time and training. A study by Acton et al. showed an 86% increase in faculty load between the academic year 2006 and 2010 which was largely attributed to implementing a new simulation curriculum as well as participating in multi-institutional simulation-based research projects [16]. Similarly, a study on simulation–based education in EM postgraduate training programs in Canada found that faculty time and training were the major obstacles to simulation implementation [17].

Models for implementing successful SBME curricula in faculty rich environments have been previously described [18]. Dagnone et al. discussed the importance of supporting faculty simulation champions who received supported education time and trained additional faculty over several years to assist in simulation instruction when developing comprehensive SBME courses [19], however this can be challenging in environments with limited faculty resources. Takahashi et al. revealed a positive correlation between the number of simulation faculty and the degree of simulation-based education implementation [20].

Little has been described regarding strategies to implement SBME curricula in programs with limited faculty resources. This posed a significant barrier to pilot curriculum development, implementation and sustainability at our site and would likely be a barrier to those wishing to incorporate SBME into their own resource limited programs. Among suggested solutions for reducing faculty time spent on simulation is using a shared case banks for simulation curricula [1]. However, even when provided cases, our faculty spent an average of 2 h to prepare for a given scenario each month, including time to program the scenario, test run the equipment, gather supplies and review relevant updates in clinical guidelines. If the curriculum was to be evaluated as we did, an additional hour of faculty time was needed to distribute, collect, and analyze evaluation data for each session. For each 2-h block of simulation teaching time, faculty found that they dedicated close to 5 h of total teaching time even with the use of pre-existing cases: 2 h were spent to prepare for the simulation event, 2 h to facilitate the simulation session, and 1 h to complete evaluation. This significant time commitment should be accounted for when programs begin incorporating SBME elements into their existing curricula.

For those educators entirely new to simulation, SBME also requires significant faculty onboarding given the complexity and variability of both high and low technology simulators and the many established methods of participant debriefing. Even with Dagnone et al.’s faculty champions, the curriculum described required 2–3 faculty a year and the time support to attend weeklong training sessions at simulation centers of excellence. This model required 6 years to fully develop and is not feasible for most residency programs who desire to add SBME to their existing education curricula.

For those programs with limited educator time, we found that incorporating SBME into pre-existing education conference time greatly increased curricular feasibility. By adapting existing weekly resident education conferences to include SBME, we were able to increase resident engagement with the curriculum, reduce time for curriculum administration activities (i.e. scheduling) and capitalize on previously protected faculty time. For those programs struggling to increase protected educator hours, identifying opportunities to replace didactic or small group learning with experiential learning could increase SBME without requiring faculty to come in during non-clinical hours.

Another way to reduce faculty time for SBME is to reduce simulation activity set-up time. The availability of a simulation technician or education coordinator who will help prepare and set up the sessions can then free up the faculty’s time making her/him more available to focus on the teaching. Educators can also plan to run a scenario several times in a row to take advantage of preparation time. In our revised curriculum, this meant shortening the simulation-based activities (40-min each) to allow for more repetitions [3] so that all residents could rotate through a simulated experience on a given day. In addition, while one group was undergoing the simulation, the 2 others did other educational activities, so the educational experience was maximized.

Given the desirability of simulation-based curricula and the time-intensive nature of SBME, we believe that more recommendations are needed to help simulation-based educators advocate for appropriate protected time. Basic guidelines regarding educator preparation time for simulation-based activities could help programs create more feasible simulation curricula. Experienced simulation-based educators should include anticipated faculty time when publishing SBME curricula or cases to help set realistic expectations. Finally, the development of needs assessment tools to identify relevant equipment, personnel, time, and space could provide educators with a more accurate picture of available resources.

## Conclusion

Given that SBME is becoming a common component of EM training programs worldwide, more information is needed on how to begin SBME incorporation and SBME feasibility in resource limited settings. Faculty educator time represents one of the scarcest resources of the resource-intensive simulation teaching modality. Our experience illustrates the challenges to implementing SBME in a low simulation resource setting. We propose protecting educator time and understanding the resources available in order to facilitate the creation of feasible curricula and hope that our experience encourages programs to adopt SBME components into their own curricula.

## Supplementary Information

**Additional file 1: Appendix A.** Needs Assessment Surveys.

**Additional file 2: Appendix B.** Resident goals and learning objectives.

**Additional file 3: Appendix C.** Simulation Sessions Evaluation Form.

**Additional file 4: Appendix D.** End of year evaluation.

**Additional file 5: Appendix E.** Revised Curriculum Evaluations.

## Data Availability

The datasets used and/or analyzed during the current study are available from the corresponding author on reasonable request.

## Abbreviations

SBME: Simulation based medical education
EM: Emergency Medicine
AUB: American University of Beirut
ACGME: Accreditation Council for Graduate Medical Education
PGY: Postgraduate year
ACLS: Advanced Cardiovascular Life Support
ATLS: Advanced Trauma Life Support
CPR: Cardiopulmonary resuscitation
RSI: Rapid sequence intubation
Min: Minute
