# Supplementary material for: Simulation-based curriculum development: lessons learnt in Global Health education
Source: BMC Med Educ. 2021 Jan 7;21:33. doi: 10.1186/s12909-020-02430-9 (PMC7792073; doi:10.1186/s12909-020-02430-9)
Supplement: Supplementary file 1 — Additional file 1: Appendix A. Needs Assessment Surveys. [file 12909_2020_2430_MOESM1_ESM.doc]

**Appendix A: Needs Assessment Surveys**

**Faculty Survey**

1. Please describe your Emergency Medicine training background

⎕ Graduated from an ACGME accredited Emergency Medicine residency program

⎕ Graduated from a NON-ACGME accredited Emergency Medicine residency program

⎕ Graduated from a non-Emergency Medicine ACGME accredited program

⎕ Graduated from a non-Emergency Medicine, non-ACGME accredited program

1. Please rate your confidence in the senior EM residents' skill (PGY 3/4) assessing and managing each clinical scenario listed below for adult patients:

- Unstable Penetrating Trauma (stab, GSW)

1. Not at all Confident (2) Slightly confident (3) Somewhat confident (4) Fairly confident (5) Extremely Confident

- Unstable Blunt Trauma

1. Not at all Confident (2) Slightly confident (3) Somewhat confident (4) Fairly confident (5) Extremely Confident

- Status Epilepticus

1. Not at all Confident (2) Slightly confident (3) Somewhat confident (4) Fairly confident (5) Extremely Confident

- Respiratory Distress

1. Not at all Confident (2) Slightly confident (3) Somewhat confident (4) Fairly confident (5) Extremely Confident

- Symptomatic Bradycardia

1. Not at all Confident (2) Slightly confident (3) Somewhat confident (4) Fairly confident (5) Extremely Confident

- Undifferentiated Hypotension

1. Not at all Confident (2) Slightly confident (3) Somewhat confident (4) Fairly confident (5) Extremely Confident

- Altered Mental Status/Lethargy

1. Not at all Confident (2) Slightly confident (3) Somewhat confident (4) Fairly confident (5) Extremely Confident

- Acute Myocardial Infarction

1. Not at all Confident (2) Slightly confident (3) Somewhat confident (4) Fairly confident (5) Extremely Confident

- Cardiac Arrest

1. Not at all Confident (2) Slightly confident (3) Somewhat confident (4) Fairly confident (5) Extremely Confident

- Supraventricular Tachycardia

1. Not at all Confident (2) Slightly confident (3) Somewhat confident (4) Fairly confident (5) Extremely Confident

- Acute Stroke

1. Not at all Confident (2) Slightly confident (3) Somewhat confident (4) Fairly confident (5) Extremely Confident

- Sepsis

1. Not at all Confident (2) Slightly confident (3) Somewhat confident (4) Fairly confident (5) Extremely Confident
2. Please rate your confidence in the senior EM residents' (PGY 3/4) skill performing each of the procedures listed:

- ACLS- Adult Resuscitation

1. Not at all Confident (2) Slightly confident (3) Somewhat confident (4) Fairly confident (5) Extremely Confident

- ATLS- Trauma Resuscitation

1. Not at all Confident (2) Slightly confident (3) Somewhat confident (4) Fairly confident (5) Extremely Confident

- Intubation

1. Not at all Confident (2) Slightly confident (3) Somewhat confident (4) Fairly confident (5) Extremely Confident

- Bag Valve Mask Ventilation

1. Not at all Confident (2) Slightly confident (3) Somewhat confident (4) Fairly confident (5) Extremely Confident

- Central Line Placement

1. Not at all Confident (2) Slightly confident (3) Somewhat confident (4) Fairly confident (5) Extremely Confident

- Arterial Line Placement

1. Not at all Confident (2) Slightly confident (3) Somewhat confident (4) Fairly confident (5) Extremely Confident

- Intraosseous Line Placement

1. Not at all Confident (2) Slightly confident (3) Somewhat confident (4) Fairly confident (5) Extremely Confident

- Pericardiocentesis

1. Not at all Confident (2) Slightly confident (3) Somewhat confident (4) Fairly confident (5) Extremely Confident

- Chest Tube Placement

1. Not at all Confident (2) Slightly confident (3) Somewhat confident (4) Fairly confident (5) Extremely Confident

- Transvenous Pacer Placement

1. Not at all Confident (2) Slightly confident (3) Somewhat confident (4) Fairly confident (5) Extremely Confident

- Electric Cardioversion/Defibrillation

1. Not at all Confident (2) Slightly confident (3) Somewhat confident (4) Fairly confident (5) Extremely Confident
2. Please rank the following categories in order of importance (first most important).  What skills are most important to improve junior EM resident (PGY 1/2) clinical performance in your department?

|  | 1 | 2 | 3 | 4 | 5 | 6 | 7 | 8 |
| --- | --- | --- | --- | --- | --- | --- | --- | --- |
| More individualized teaching |  |  |  |  |  |  |  |  |
| More practice taking care of acutely ill/critical care pediatric patients |  |  |  |  |  |  |  |  |
| More practice with resuscitation team leadership |  |  |  |  |  |  |  |  |
| More practice with procedures |  |  |  |  |  |  |  |  |
| More feedback on resuscitation of acutely ill/critical care patients |  |  |  |  |  |  |  |  |
| More practice building differential diagnosis for patient presentations |  |  |  |  |  |  |  |  |
| More practice taking care of acutely ill/critical care adult patients |  |  |  |  |  |  |  |  |
| More practice with communication skills in the clinical area |  |  |  |  |  |  |  |  |

1. Please rank the following categories in order of importance (first most important).  What skills are most important to improve senior EM resident (PGY 3/4) clinical performance in your department?

|  | 1 | 2 | 3 | 4 | 5 | 6 | 7 | 8 |
| --- | --- | --- | --- | --- | --- | --- | --- | --- |
| More practice with procedures |  |  |  |  |  |  |  |  |
| More individualized teaching |  |  |  |  |  |  |  |  |
| More practice building differential diagnosis for patient presentations |  |  |  |  |  |  |  |  |
| More feedback on resuscitation of acutely ill/critical care patients |  |  |  |  |  |  |  |  |
| More practice taking care of acutely ill/critical care pediatric patients |  |  |  |  |  |  |  |  |
| More practice taking care of acutely ill/critical care adult patients |  |  |  |  |  |  |  |  |
| More practice with communication skills in the clinical area |  |  |  |  |  |  |  |  |
| More practice with resuscitation team leadership |  |  |  |  |  |  |  |  |

1. Please list three procedures you believe the residents need more practice on as part of their Emergency Medicine training

1.

2.

3.

1. Are there any areas you believe junior EM residents (i.e. PGY 1, PGY 2) could receive additional training in that would benefit their education during residency?

__________________________________________________________________________________________________________________________________________________________________________________________________

1. Are there any areas you believe senior EM residents (i.e. PGY 3, PGY 4) could receive additional training in that would benefit their education during residency?

__________________________________________________________________________________________________________________________________________________________________________________________________

1. Please provide any other comments/feedback you would like to include to inform the creation of a simulation-based educational curriculum to complement the existing educational curriculum at the Emergency Medicine Residency Program

__________________________________________________________________________________________________________________________________________________________________________________________________

1. Would you be willing to be trained in facilitating simulation-based educational activities so that you could run simulations during your educational conference teaching time?

Yes No I don't teach at conference

**Residents Survey**

1. What is your PGY year? ⎕PGY1 ⎕PGY2 ⎕ PGY3 ⎕ PGY4
2. Please list the three most common causes of morbidity/mortality among patients that present to your Emergency Department

1.

2.

3.

1. Please list the three concepts or skills you would like to learn more about in order to be more effective in managing critically ill patients in your Emergency Department

1.

2.

3.

1. Please list three procedures you would like to practice more often in your EM training

1.

2.

3.

1. Please rate your confidence in assessing and managing each clinical scenario listed below for adult patients

- Acute Myocardial Infarction

1. Not at all Confident (2) Slightly confident (3) Somewhat confident (4) Fairly confident (5) Extremely Confident

- Undifferentiated Hypotension

1. Not at all Confident (2) Slightly confident (3) Somewhat confident (4) Fairly confident (5) Extremely Confident

- Sepsis

1. Not at all Confident (2) Slightly confident (3) Somewhat confident (4) Fairly confident (5) Extremely Confident

- Cardiac Arrest

1. Not at all Confident (2) Slightly confident (3) Somewhat confident (4) Fairly confident (5) Extremely Confident

- Respiratory Distress

1. Not at all Confident (2) Slightly confident (3) Somewhat confident (4) Fairly confident (5) Extremely Confident

- Symptomatic Bradycardia

1. Not at all Confident (2) Slightly confident (3) Somewhat confident (4) Fairly confident (5) Extremely Confident

- Supraventricular Tachycardia

1. Not at all Confident (2) Slightly confident (3) Somewhat confident (4) Fairly confident (5) Extremely Confident

- Status Epilepticus

1. Not at all Confident (2) Slightly confident (3) Somewhat confident (4) Fairly confident (5) Extremely Confident

- Altered Mental Status/Lethargy

1. Not at all Confident (2) Slightly confident (3) Somewhat confident (4) Fairly confident (5) Extremely Confident

- Acute Stroke

1. Not at all Confident (2) Slightly confident (3) Somewhat confident (4) Fairly confident (5) Extremely Confident

- Unstable Blunt Trauma

1. Not at all Confident (2) Slightly confident (3) Somewhat confident (4) Fairly confident (5) Extremely Confident

- Unstable Penetrating Trauma (stab, GSW)

1. Not at all Confident (2) Slightly confident (3) Somewhat confident (4) Fairly confident (5) Extremely Confident
2. Please rate your confidence in performing each of the procedures listed

- ACLS- Adult Resuscitation

1. Not at all Confident (2) Slightly confident (3) Somewhat confident (4) Fairly confident (5) Extremely Confident

- ATLS- Trauma Resuscitation

1. Not at all Confident (2) Slightly confident (3) Somewhat confident (4) Fairly confident (5) Extremely Confident

- Intubation

1. Not at all Confident (2) Slightly confident (3) Somewhat confident (4) Fairly confident (5) Extremely Confident

- Bag Valve Mask Ventilation

1. Not at all Confident (2) Slightly confident (3) Somewhat confident (4) Fairly confident (5) Extremely Confident

- Central Line Placement

1. Not at all Confident (2) Slightly confident (3) Somewhat confident (4) Fairly confident (5) Extremely Confident

- Arterial Line Placement

1. Not at all Confident (2) Slightly confident (3) Somewhat confident (4) Fairly confident (5) Extremely Confident

- Intraosseous Line Placement

1. Not at all Confident (2) Slightly confident (3) Somewhat confident (4) Fairly confident (5) Extremely Confident

- Pericardiocentesis

1. Not at all Confident (2) Slightly confident (3) Somewhat confident (4) Fairly confident (5) Extremely Confident

- Chest Tube Placement

1. Not at all Confident (2) Slightly confident (3) Somewhat confident (4) Fairly confident (5) Extremely Confident

- Transvenous Pacer Placement

1. Not at all Confident (2) Slightly confident (3) Somewhat confident (4) Fairly confident (5) Extremely Confident

- Electric Cardioversion/Defibrillation

1. Not at all Confident (2) Slightly confident (3) Somewhat confident (4) Fairly confident (5) Extremely Confident
2. We are developing a simulation-based component to your residency educational curriculum.  Please rank your preference for the following simulation based educational activity options

- Individual 1-2 hour modules (1 resident with a faculty member) outside of conference time
- Small group 1-2 hour modules (2-4 residents with a faculty member) outside of conference time
- Simulation incorporated into conference teaching (up to 8-10 residents in a group)

1. Would you be willing to come in during time off (outside of conference time) to participate in simulation-based educational activities?

⎕Yes ⎕No

1. Please rank the following categories on what is most important to improve the educational experience in your residency program

|  | 1 | 2 | 3 | 4 | 5 | 6 | 7 | 8 |
| --- | --- | --- | --- | --- | --- | --- | --- | --- |
| More practice with procedures |  |  |  |  |  |  |  |  |
| More practice taking care of acutely ill/critical care adult patients |  |  |  |  |  |  |  |  |
| More practice building differential diagnosis for patient presentations |  |  |  |  |  |  |  |  |
| More feedback on your resuscitation of acutely ill/critical care patients |  |  |  |  |  |  |  |  |
| More practice taking care of acutely ill/critical care pediatric patients |  |  |  |  |  |  |  |  |
| More practice with resuscitation team leadership |  |  |  |  |  |  |  |  |
| More practice with communication skills in the clinical area |  |  |  |  |  |  |  |  |

1. Please provide any other comments/feedback you would like to include to inform the simulation curriculum development for your residency program

___________________________________________________________________________________________________________________________________________________________________________________________________________________________________________________________________________________________________________________________
