# Supplementary material for: Simulation-based curriculum development: lessons learnt in Global Health education
Source: BMC Med Educ. 2021 Jan 7;21:33. doi: 10.1186/s12909-020-02430-9 (PMC7792073; doi:10.1186/s12909-020-02430-9)
Supplement: Supplementary file 2 — Additional file 2: Appendix B. Resident goals and learning objectives. [file 12909_2020_2430_MOESM2_ESM.doc]

**Appendix B: Resident goals and learning objectives**

PGY-I Goals:

1. Introduction to the simulation environment and to simulated patient management skills.
2. Demonstrate respect and compassion for patients.
3. Demonstrate effective communication skills with patients, families, and team members.
4. Demonstrate effective application of medical knowledge to clinical situations.
5. Observe self- and peers performance/skills/attitudes and provide effective self/other-directed feedback.

PGY1: Objectives

By the end of PGY-1, junior residents will demonstrate:

- a systematic approach to history and physical exam that they will apply to building a differential diagnosis for each presentation.
- appropriate assessments and management plans based on their differential diagnosis.
- the ability to use AHA algorithms for simple patient resuscitations.

PGY II Goals:

1. Successful management of more complex medical cases and equipment failures.
2. Analysis and improvement of systems performance as well as individual performance.
3. Demonstrate respect and compassion for a diverse patient population.
4. Demonstrate effective communication skills with patients, families, and team members in more challenging environments.
5. Observe own performance/skills/attitudes and team members’ performance/skills/attitudes and provide effective self/other-directed feedback.

PGYII: Objectives

By the end of PGY-2, the resident will demonstrate:

- a systematic approach to history and physical exam that they will apply to building a differential diagnosis for complex medical presentations.
- an understanding of their role in the emergency department system and team
- effective communication within the medical team

be able to apply the lessons learnt in simulation on complex cases in the emergency department on complex cases. S/he will be able to understand their role in the emergency department system and team. Simulations will continue focusing on medical knowledge of more complex cases and will help the resident understand and apply effective communication skills within a medical team.

PGY III Goals:

1. Continued analysis and improvement of systems performance as well as individual performance.
2. Demonstrate adequate management of complex medical cases and equipment failures.
3. Demonstrate a high level of sophistication in interpersonal skills, professionalism, and interaction in complex medical system.
4. Simultaneously manage multiple patients in a realistic emergency department setting.
5. Develop personal strategies for self-improvement, error reduction, and lifelong learning.

PGYIII: Objectives

By the end of PGY-3, the resident will be able analyze and evaluate their performance during scenarios and apply the knowledge in the emergency department. Their simulation education will focus on team communications and interpersonal skills. Scenarios will be more complex including multiple patients e.g precipitous delivery.

By the end of PGY-3, the senior resident will demonstrate:

- the ability to manage pediatric and neonatal critical care patients requiring resuscitation
- the ability to evaluate and analyze their own performance and show self-improvement
- effective team communication and interpersonal skills
- the ability to lead the medical team in medical resuscitation

PGY IV goals:

1. Demonstrate adequate leadership roles in medical and surgical resuscitations.
2. Apply evidence-based medicine in medical decisions making and practice.
3. Demonstrate a high level of sophistication in interpersonal skills, professionalism, and interaction in complex medical system.
4. Adequate use of resources, protocols, and crisis management.
5. Adequately deal with complex ethical and system-based challenges.

PGY IV objectives:

By the end of PGY-4, the resident will be able analyze and evaluate the whole team’s performance during scenarios and provide feedback that will be applied in the emergency department. They will apply crisis resource management principles and deal with complex system and patient problems.

By the end of PGY-4, the resident will demonstrate:

- correct use of the difficult airway algorithm for management of difficult and failed airways
- the ability to apply crisis resource management principles to lead complex critical care scenarios
- the ability to lead the medical team in complex medical resuscitation
- the ability to employ flexible communication strategies to deal with difficult consultants, patients and family members.
