# Supplementary material for: Simulation-based curriculum development: lessons learnt in Global Health education
Source: BMC Med Educ. 2021 Jan 7;21:33. doi: 10.1186/s12909-020-02430-9 (PMC7792073; doi:10.1186/s12909-020-02430-9)
Supplement: Supplementary file 3 — Additional file 3: Appendix C. Simulation Sessions Evaluation Form. [file 12909_2020_2430_MOESM3_ESM.doc]

**Appendix C: Simulation Sessions Evaluation Form**

**Simulation Evaluation Form**

PGY level: _____ Date: __________


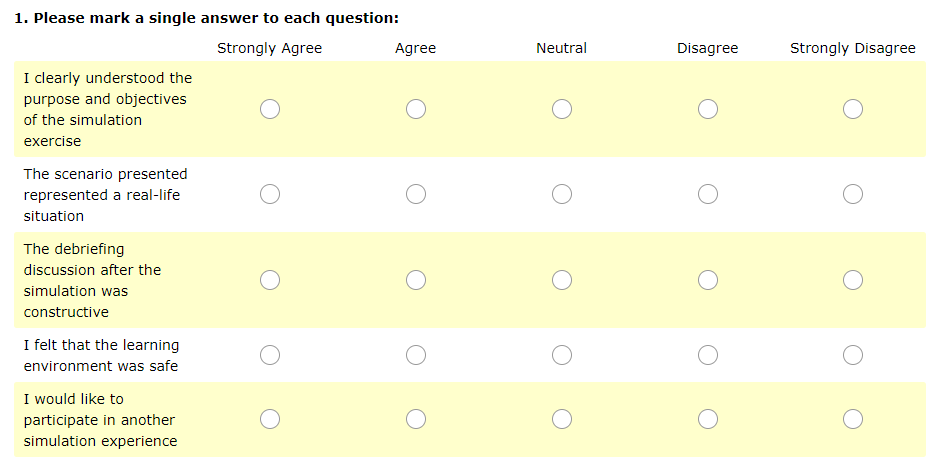


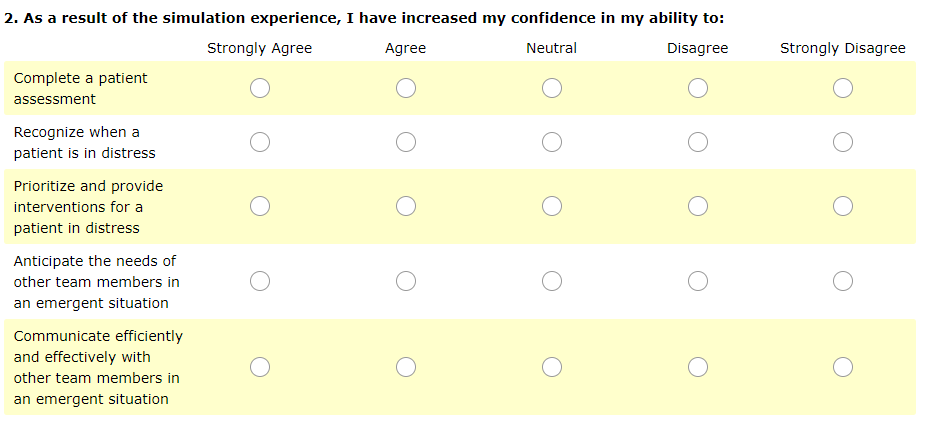


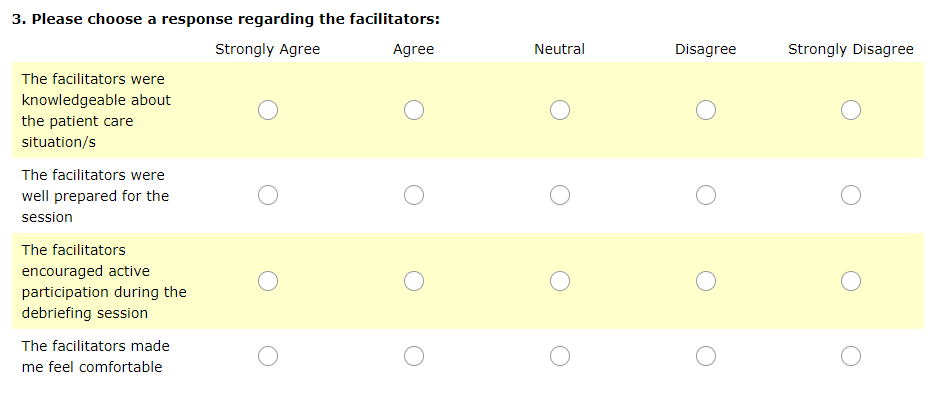


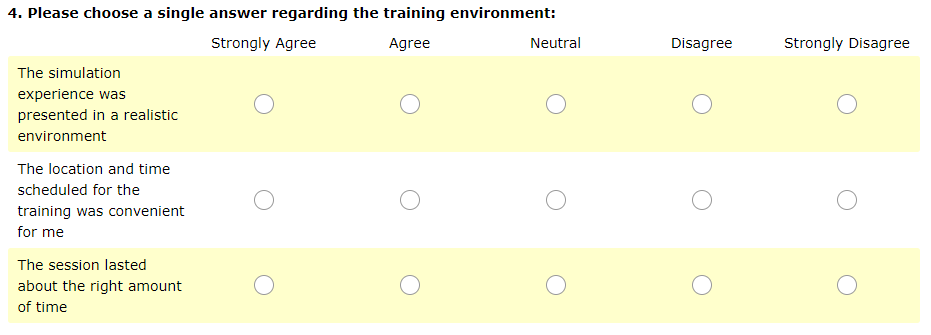


**5. What changes would you recommend to improve future simulation experiences?**

**__________________________________________________________________________________________________________________________________________________________________________________________________________________________________________**
