# Supplementary material for: Simulation-based curriculum development: lessons learnt in Global Health education
Source: BMC Med Educ. 2021 Jan 7;21:33. doi: 10.1186/s12909-020-02430-9 (PMC7792073; doi:10.1186/s12909-020-02430-9)
Supplement: Supplementary file 4 — Additional file 4: Appendix D. End of year evaluation. [file 12909_2020_2430_MOESM4_ESM.doc]

**Appendix D: End of year evaluation**

**Simulation Curriculum Evaluation Survey**

*This is an anonymous survey for you to evaluate the simulation curriculum which you participated in during 2018-2019 academic year. We would like your feedback to help us improve this curriculum in the future. This should take about 5 minutes or less for you to complete. Your responses will in no way impact your current or future educational opportunities. Thank you for your candid feedback.*

Current PGY level: II III IV

**Please answer these questions based on your experience during the past 2018-2019 simulation sessions**

Do you feel that the simulation curriculum increased your confidence with the management of the following scenarios?

- Pediatric septic shock

1. Strongly agree (2) Agree (3) Neutral (4) Disagree (5) Strongly disagree

- Status epilepticus

1. Strongly agree (2) Agree (3) Neutral (4) Disagree (5) Strongly disagree

- Hyperkalemia

1. Strongly agree (2) Agree (3) Neutral (4) Disagree (5) Strongly disagree

- Pediatric respiratory distress

1. Strongly agree (2) Agree (3) Neutral (4) Disagree (5) Strongly disagree

- Cardiac arrest

1. Strongly agree (2) Agree (3) Neutral (4) Disagree (5) Strongly disagree

- Unstable blunt trauma

1. Strongly agree (2) Agree (3) Neutral (4) Disagree (5) Strongly disagree

- Acute myocarditis

1. Strongly agree (2) Agree (3) Neutral (4) Disagree (5) Strongly disagree

- Intoxication

1. Strongly agree (2) Agree (3) Neutral (4) Disagree (5) Strongly disagree

- Electrocution

1. Strongly agree (2) Agree (3) Neutral (4) Disagree (5) Strongly disagree

Do you feel that the simulation curriculum increased your confidence with the following procedures?

- Endotracheal intubation

1. Strongly agree (2) Agree (3) Neutral (4) Disagree (5) Strongly disagree

- Cardiopulmonary resuscitation (CPR)

1. Strongly agree (2) Agree (3) Neutral (4) Disagree (5) Strongly disagree

- Pericardiocentesis

1. Strongly agree (2) Agree (3) Neutral (4) Disagree (5) Strongly disagree

- Defibrillation/cardioversion

1. Strongly agree (2) Agree (3) Neutral (4) Disagree (5) Strongly disagree

- Primary survey

1. Strongly agree (2) Agree (3) Neutral (4) Disagree (5) Strongly disagree

- Secondary survey

1. Strongly agree (2) Agree (3) Neutral (4) Disagree (5) Strongly disagree

- Communication skills in the clinical area

1. Strongly agree (2) Agree (3) Neutral (4) Disagree (5) Strongly disagree

- Role assignment/recognition

1. Strongly agree (2) Agree (3) Neutral (4) Disagree (5) Strongly disagree

Where you the leader in at least 1 simulation scenario? ◌ Y ◌ N

If yes, did the simulation increase your resuscitation leadership skills?

1. Strongly agree (2) Agree (3) Neutral (4) Disagree (5) Strongly disagree

Do you feel that you need more practice with the following skills?

- Procedures

1. Strongly agree (2) Agree (3) Neutral (4) Disagree (5) Strongly disagree

Please specify which:

- Taking care of acutely ill/critically ill adult patients

(1) Strongly agree (2) Agree (3) Neutral (4) Disagree (5) Strongly disagree

- Taking care of acutely ill/critically ill pediatric patients

(1) Strongly agree (2) Agree (3) Neutral (4) Disagree (5) Strongly disagree

- Building differential diagnosis for patient presentations

1. Strongly agree (2) Agree (3) Neutral (4) Disagree (5) Strongly disagree

- Closed loop communication

1. Strongly agree (2) Agree (3) Neutral (4) Disagree (5) Strongly disagree

- Role assignment/recognition

1. Strongly agree (2) Agree (3) Neutral (4) Disagree (5) Strongly disagree

- Resuscitation team leadership

1. Strongly agree (2) Agree (3) Neutral (4) Disagree (5) Strongly disagree

What are the strengths of the current simulation curriculum?

__________________________________________________________________________________________________________________________________________________________________________________________________________________________________________

What changes do you suggest making to improve the simulation curriculum?

__________________________________________________________________________________________________________________________________________________________________________________________________________________________________________
