# Supplementary material for: Simulation-based curriculum development: lessons learnt in Global Health education
Source: BMC Med Educ. 2021 Jan 7;21:33. doi: 10.1186/s12909-020-02430-9 (PMC7792073; doi:10.1186/s12909-020-02430-9)
Supplement: Supplementary file 5 — Additional file 5: Appendix E. Revised Curriculum Evaluations. [file 12909_2020_2430_MOESM5_ESM.doc]

**Appendix E : Revised Curriculum Evaluations**

# Individual simulation modules’ evaluation:

On a 5-point Likert scale (with 1 being strongly agree and 5 being strongly disagree), residents rated questions related to the individual simulation experience, their perceived increased confidence with behavioral skills, facilitators performance, and the training environment. In addition, we requested feedback by free text comments.

We collected 91 evaluations from all participants (12-15 participant/session) over the eight simulation activities that were performed during the revised curriculum phase (year 2). The evaluation form of the first simulation session did not include the variable of PGY level so the results of this session were excluded from our analysis (a total of 14 responses). The modules with the highest total average scores include: Ventricular Fibrillation Secondary to hyperkalemia (score 1.01) and Bronchiolitis (score 1.03) (see Table 1). In addition, the average score for each question across all eight sessions was calculated. Results showed that, on average, residents rated most of the questions highly. The highest score (1.07 for all 3) was given to the following: facilitators were well prepared for the session, the learning environment was safe, and residents would like to participate in another simulation experience. On the other hand, the questions which received the lowest scores related to increased confidence in recognizing a patient in distress and doing a patient assessment, albeit still at 1.34 and 1.31 respectively. When analyzed by PGY level, these latter 2 questions were the only ones which showed a statistically significant (p<0.05) difference between trainee level where confidence increased significantly more in PGY1 compared to PGY4 residents (see table 2).

| **Module Title** | **Total Average Score (of 17 questions) Per Module** |
| --- | --- |
| 1.       Sim 1- Pediatric Septic Shock | 1.39 |
| 2.       Sim 2 -Status Epilepticus | 1.12 |
| 3.       Sim 3 - Ventricular Fibrillation Secondary to hyperkalemia | 1.01 |
| 4.       Sim 4- Bronchiolitis (pediatric) | 1.03 |
| 5.       Sim 5-Cardiac Arrest | 1.11 |
| 6.       Sim 6- Trauma Resuscitation | 1.34 |
| 7.       Sim 7 - Acute Myocarditis (pediatric) | 1.09 |
| 8. Sim 8- Organophosphate Poisoning | 1.05 |

**Table 1: Total average score per Module (1 is strongly agree; 5 is least agree)**

| **Question** | **PGY Level** | **Number of Responses** | **Mean** | **P Value** |
| --- | --- | --- | --- | --- |
|  |  |  |  |  |
| The scenario presented represented a real-life situation | 1 | 17 | 1.00 | 0.453 |
|  | 2 | 21 | 1.00 |  |
|  | 3 | 21 | 1.10 |  |
|  | 4 | 18 | 1.00 |  |
|  | Total | 77 | **1.03** |  |
| I clearly understood the purpose and objectives of the simulation exercise | 1 | 17 | 1.00 | 0.509 |
|  | 2 | 21 | 1.05 |  |
|  | 3 | 21 | 1.14 |  |
|  | 4 | 18 | 1.06 |  |
|  | Total | 77 | **1.06** |  |
| The debriefing discussion after the simulation was constructive | 1 | 17 | 1.00 | 0.579 |
|  | 2 | 21 | 1.00 |  |
|  | 3 | 21 | 1.10 |  |
|  | 4 | 18 | **1.06** |  |
|  | Total | 77 | 1.04 |  |
| I felt that the learning environment was safe | 1 | 17 | 1.00 | 0.060 |
|  | 2 | 21 | 1.00 |  |
|  | 3 | 21 | 1.19 |  |
|  | 4 | 18 | 1.00 |  |
|  | Total | 77 | **1.05** |  |
| I would like to participate in another simulation experience | 1 | 17 | 1.00 | 0.579 |
|  | 2 | 21 | 1.00 |  |
|  | 3 | 21 | 1.10 |  |
|  | 4 | 18 | 1.06 |  |
|  | Total | 77 | **1.04** |  |
| As a result of the simulation experience, I have increased my confidence in my ability to: complete a patient assessment | 1 | 17 | 1.06 | 0.021 |
|  | 2 | 21 | 1.10 |  |
|  | 3 | 21 | 1.43 |  |
|  | 4 | 18 | 1.50 |  |
|  | Total | 77 | **1.27** |  |
| As a result of the simulation experience, I have increased my confidence in my ability to: recognize when a patient is in distress | 1 | 17 | 1.06 | 0.025 |
|  | 2 | 21 | 1.05 |  |
|  | 3 | 21 | 1.48 |  |
|  | 4 | 18 | 1.39 |  |
|  | Total | 77 | **1.25** |  |
| As a result of the simulation experience, I have increased my confidence in my ability to: prioritize and provide interventions for a patient in distress | 1 | 17 | 1.12 | 0.208 |
|  | 2 | 21 | 1.05 |  |
|  | 3 | 21 | 1.24 |  |
|  | 4 | 18 | 1.33 |  |
|  | Total | 77 | **1.18** |  |
| As a results of the simulation experience, I have increased my confidence in my ability to: anticipate the needs of other team members in an emergency situation | 1 | 17 | 1.06 | 0.108 |
|  | 2 | 21 | 1.05 |  |
|  | 3 | 21 | 1.29 |  |
|  | 4 | 18 | 1.28 |  |
|  | Total | 77 | **1.17** |  |
| As a result of the simulation experience, I have increased my confidence in my ability to: communicate efficiently and effectively with other team members in an emergency situation. | 1 | 17 | 1.06 | 0.539 |
|  | 2 | 21 | 1.14 |  |
|  | 3 | 21 | 1.24 |  |
|  | 4 | 18 | 1.22 |  |
|  | Total | 77 | **1.17** |  |
| The facilitators were knowledgeable about the patient care situation/s | 1 | 17 | 1.00 | 0.479 |
|  | 2 | 21 | 1.05 |  |
|  | 3 | 20 | 1.15 |  |
|  | 4 | 18 | 1.06 |  |
|  | Total | 76 | **1.07** |  |
| The facilitators were well prepared for the session | 1 | 17 | 1.00 | 0.760 |
|  | 2 | 21 | 1.05 |  |
|  | 3 | 20 | 1.10 |  |
|  | 4 | 18 | 1.06 |  |
|  | Total | 76 | **1.05** |  |
| The facilitators encouraged active participation during the debriefing session | 1 | 17 | 1.00 | 0.733 |
|  | 2 | 21 | 1.10 |  |
|  | 3 | 20 | 1.10 |  |
|  | 4 | 18 | 1.06 |  |
|  | Total | 76 | **1.07** |  |
| The facilitators made me feel comfortable | 1 | 17 | 1.00 | 0.760 |
|  | 2 | 21 | 1.05 |  |
|  | 3 | 20 | 1.10 |  |
|  | 4 | 18 | 1.06 |  |
|  | Total | 76 | **1.05** |  |
| The simulation experience was presented in a realistic environment | 1 | 17 | 1.12 | 0.260 |
|  | 2 | 21 | 1.10 |  |
|  | 3 | 20 | 1.35 |  |
|  | 4 | 18 | 1.28 |  |
|  | Total | 76 | **1.21** |  |
| The location and time scheduled for the training was convenient for me | 1 | 17 | 1.00 | 0.493 |
|  | 2 | 21 | 1.10 |  |
|  | 3 | 20 | 1.15 |  |
|  | 4 | 18 | 1.17 |  |
|  | Total | 76 | **1.11** |  |
| The session lasted about the right amount of time | 1 | 17 | 1.00 | 0.063 |
|  | 2 | 21 | 1.14 |  |
|  | 3 | 20 | 1.20 |  |
|  | 4 | 18 | 1.50 |  |
|  | Total | 76 | **1.21** |  |

**Table 2: Average evaluation scores for all 8 modules, stratified by level of training**

Residents’ comments after each simulation module were also analyzed. We report the comments which provided feedback on improving the simulation experience itself. Three themes were prevailed across all respondents (see table 3). The first most frequent theme is that residents requested more realistic scenarios, followed by allocating more time for the modules, and finally to increase the frequency of simulation sessions.

| **Things done well** | **Areas for improvement** |
| --- | --- |
| - Very smooth and educational (1) - Sim sessions are getting more and more realistic and useful, translating to my performance in Emergency Department with actual patients (1) - Amazing session (1) - Thank you for all the effort you make for us (1) - Debrief is very helpful (2) | - Make it more real - Printout physical exam findings or make them easier to identify (2) - Have less residents per session (2) - Add more procedures (2) - Use video debrief (1) - Increase the frequency of sim sessions (7) - Include nursing team in sessions (4) - Increase the time for discussion (1) - Make the timing more realistic e.g say how much time has passed (1) - Encourage participants to slow down (1) - Make injuries more visible/obvious (2). - Have a dedicated individual acting as the patient to respond to questions and react to the physical exam performed on the mannequin (1) - Have time for repeating the simulation scenario after debriefing scenario - Ultrasound at bedside (1) - Longer sessions (2) - Better coordination between facilitators (1) - Put the same level residents on the same group. - Get family members/EMS to get history (2). |

**Table 3: Open text feedback on individual simulation modules**

(x): number of times the comment was provided

# Whole revised curriculum evaluation:

On a 5 point Likert scale (with 1 being strongly agree and 5 being strongly disagree), residents rated questions related to the effect of simulation curriculum on their confidence in managing certain scenarios and procedures, on their resuscitation and team leadership skills, and on whether they felt that they needed more practice on specific skills. In addition, we requested feedback by free text comments.

We collected 14/16 responses from residents who participated in the sessions (response rate was 87.5%); PGY 4 residents who graduated from the program where excluded as we could not contact them. When residents did not attend a specific session, they answered “not applicable”.

When asked whether the specific simulation scenarios improved their confidence in managing certain scenarios, residents felt most confident with cardiac arrest scenarios (average score 1.57) and least confident with status epilepticus scenarios (average score 2.46). With respect to procedures, residents felt that they became most confident in role assignment/recognition (average score 1.57) but remained least confident in performing pericardiocentesis (average score 2.67). All residents had the chance to lead the management of at least one simulation scenario. However, when asked about whether simulation increased their resuscitation team leadership, 64% answered “No”. In addition, residents felt that they needed to practice procedures in general the least (average score= 1.46) but needed more training generating a differential diagnosis (average score= 2.69). Some of the procedures that residents requested more practice included pericardiocentesis, endotracheal intubations and Cardiopulmonary resuscitation, cardioversion, and sedation.

Of note, we stratified the answers by PGY level but did not find any statistical difference.

| **Question:** | **Total average score across all PGY levels** |
| --- | --- |
| Do you feel that the simulation curriculum helped you increasing your confidence with the management of the following scenarios? | |
| Status epilepticus | 2.46 |
| Hyperkalemia | 2.46 |
| Pediatric septic shock | 2.36 |
| Intoxication | 2.33 |
| Pediatric respiratory distress | 2.14 |
| Unstable blunt trauma | 2.08 |
| Acute myocarditis | 2.00 |
| Electrocution | 2.00 |
| Cardiac arrest | 1.57 |
| Do you feel that the simulation curriculum helped you increasing your confidence with the following procedures? | |
| Pericardiocentesis | 2.67 |
| Endotracheal intubation | 2.07 |
| Cardiopulmonary resuscitation (CPR) | 2.07 |
| Defibrillation/cardioversion | 1.71 |
| Secondary survey | 1.64 |
| Primary survey | 1.57 |
| Communication skills in the clinical area | 1.57 |
| Role assignment/recognition | 1.57 |
| Do you feel that you need more practice with the following skills? | |
| Building differential diagnosis for patient presentations | 2.69 |
| Taking care of acutely ill/critically ill adult patients | 2.36 |
| Closed loop communication | 2.36 |
| Resuscitation team leadership | 2.29 |
| Role assignment/recognition | 2.07 |
| Taking care of acutely ill/critically ill pediatric patients | 1.86 |
| Procedures | 1.46 |

**Table 1: Results of end of year simulation curriculum evaluation**

Residents’ free text comments were also analyzed. We report the comments which provided feedback on improving the simulation experience itself. We noticed that most of the comments fell under one main theme, which is increasing the frequency of simulation sessions and the cases presented. Residents wanted more simulations with more emphasis on both adults and pediatric cases. They also wanted to practice on uncommon procedures such as cricothyrotomy and on dosages of rapid sequence intubation (RSI)/sedation/ and pediatrics. Moreover, comments included more focus on communication skills and on medical cases with less focus on trauma. Some other suggestions included providing reading material related to the upcoming session and assigning a team leader in the group.

| **Things done well** | **Areas for improvement** |
| --- | --- |
| - Medical take home messages - Cases resemble real life situations (2) - Debriefing is educational - Feedback is practical and immediate - Dividing residents into groups according to same PGY level (2) - Cases are fun, clear, accessible, and Educational - Simulation run time is adequate - Environment is safe - I feel more comfortable regarding critical situations | - More adult cases please - More uncommon cases/procedures (3) - More pediatric cases (2) - Do it more frequently (3) - Emphasize more on debriefing and feedback. - More cases with emphasis on communication skills (difficult patients, agitated patients or family members). - More medical - Provide reading material related to the upcoming session - Pre-Assign the team leader and other roles in the group by facilitators(2) |

**Table 5: Open text feedback on individual simulation sessions**

(x): number of times the comment was provided
